# Prevalence and Correlates of Cognitive Impairment No Dementia (CIND) Status: The Midlife in the United States Study.

**DOI:** 10.1093/geroni/igaf122.2408

**Published:** 2025-12-31

**Authors:** Robert Stawski, Dakota Witzel, Eric Cerino, Stuart MacDonald

**Affiliations:** Utah State University, Logan, Utah, United States; South Dakota State University, Brookings, South Dakota, United States; Northern Arizona University, Flagstaff, Arizona, United States; University of Victoria, Victoria, British Columbia, Canada

## Abstract

Cognitive impairment no dementia (CIND) status – a multi-test profile and impairment classification – is a promising indicator of cognitive health, leveraging intraindividual variability in performance across multiple domains. CIND profiles have exhibited considerable utility for differentiating adults exhibiting neuropsychological impairment (>-1SD below age- and education-adjusted norms) on one (CIND-single) or more (CIND-multiple) tasks, from those who are comparatively unimpaired or exhibit transient low scores due to temporary factors (e.g., minor illness). Research employing CIND profiles has focused on older populations, leaving questions about the prevalence, distribution, and correlates of CIND status among midlife adults, who are at risk for dementia. Using data from the MIDUS II study (N = 4,241; Mage=55.6, SD = 12.2, Range=28-84; 55%=women), participants completed the Brief Test of Adult Cognition via Telephone (BTACT), which includes tests of executive function and episodic memory abilities. CIND status prevalences were 50.2% non-CIND, 26.8% CIND-single, and 23.0% CIND-multiple. Significantly more men (p=.03), non-White (p<.0001), and lower-educated (p<.0001) adults met CIND-single and CIND-multiple criteria. Response time inconsistency (RTI), an index of central nervous system integrity and impairment/dementia risk, was greater among CIND-single (Cohen’s d=.20, p<.01) and CIND-multiple (Cohen’s d=.52, p<.001) participants, relative to non-CIND participants. Covariate-adjusted multinomial logistic regression models revealed RTI was associated with increased odds of CIND-single (p<.006) and CIND-multiple (p<.0001) statuses. Further, gender, education, and self-rated health also exhibited independent associations indicative of increased CIND classification risk (ps<.0001). Discussion will focus on the utility of CIND status for understanding patterns and profiles of cognitive health, and early detection of dementia risk.

